# Incidence rates and temporal trends of cervical cancer relating to opportunistic screening in two developed metropolitan regions of Brazil: a population-based cohort study

**DOI:** 10.1590/1516-3180.2018.0306220719

**Published:** 2019-10-31

**Authors:** Júlio César Teixeira, Carlos Afonso Maestri, Helymar da Costa Machado, Luiz Carlos Zeferino, Newton Sérgio de Carvalho

**Affiliations:** I MD, PhD. Physician and Assistant Professor, Department of Obstetrics and Gynecology, School of Medical Sciences, Universidade Estadual de Campinas (UNICAMP), Campinas (SP), Brazil.; II MD, MSc. Physician and Doctoral Student, Lower Genital Tract Disease Service, Hospital Erasto Gaertner, Curitiba (PR), Brazil.; III MSc. Statistician, Department of Statistics, Centro de Atenção Integral à Saúde da Mulher (CAISM), Universidade Estadual de Campinas (UNICAMP), Campinas (SP), Brazil.; IV MD, PhD. Physician and Professor, Department of Obstetrics and Gynecology, School of Medical Sciences, Universidade Estadual de Campinas (UNICAMP), Campinas (SP), Brazil.; V MD, PhD. Physician and Professor, Department of Obstetrics and Gynecology, School of Medical Sciences, Universidade Federal do Paraná (UFPR), Curitiba (PR), Brazil.

**Keywords:** Uterine cervical neoplasms, Epidemiology, Public health, Early detection of cancer

## Abstract

**BACKGROUND:**

Brazilian opportunistic screening programs for cervical cancer have limited impact. In the regions of two cities (Campinas and Curitiba) with high human development indices, consistent information from 96-97% of all cervical cancer cases managed within the public healthcare system is available.

**OBJECTIVE:**

To estimate the incidence rate (IR) and temporal trends in these regions, covering 2001-2012.

**DESIGN AND SETTING:**

A population-based cohort study was conducted under the assumption that all cervical cancer cases were managed in cancer referral center hospitals.

**METHODS:**

3,364 records (1,646 from Campinas; 1,718 from Curitiba) were analyzed to provide estimates of IR, age-standardized IR (ASR) and cervical cancer trends (shown per 100,000 women/year). Longitudinal patterns were analyzed using linear regression and shown as annual percentage change (APC); P < 0.05 for significance.

**RESULTS:**

Annual IR and ASR estimates for cervical cancer ranged from 3.8 to 8.0 over 2001-2012, decreasing over more recent years, and were similar for the two regions. The age-specific IR was about 50% lower among women aged 45 years or older (IR-2001/IR-2012: Campinas = 14.8/8.0; Curitiba = 18.7/8.3; P < 0.001). There was an increasing APC trend in Campinas among women aged 15-24 years, and a decreasing IR trend for squamous-cell histology in both regions (P < 0.05).

**CONCLUSION:**

Cervical cancer incidence estimates showed slowly decreasing trends in both regions, most evidently for women aged 45 years or older and for squamous-cell histology. These findings reflect the opportunistic nature of the population screening program, despite the comparatively high economic development level in the two regions.

## INTRODUCTION

Cervical cancer remains an important burden in relation to women’s health, especially in places with limited resources. The Brazilian government estimated that there would be 16,370 new cases in 2018,^1^ with great regional variability. Thus, cervical cancer was placed as between the first and fourth most prevalent forms of cancer among women.^1^^,^^2^

Cervical cancer and its precursor lesions can be detected by screening. The success of population screening programs relates to their level of organization. Organized programs have defined target populations (age, screening interval and satisfactory access), predefined treatment algorithms, management teams, structures for quality assurance, surveillance systems, enough coverage and population education. Such programs have the potential to decrease cervical cancer rates by 80%.^3^

In contrast, there are opportunistic screening programs, such as the official population-screening program of the Brazilian National Health System (Sistema Único de Saúde, SUS). There is no registration system to control the screened or unscreened population and cytological tests are performed opportunistically, as a result of recommendations made during routine medical consultations, based on presence of increased risk of developing cancer, or through self-referral. The Brazilian program indicates that conventional cytological tests should be performed every three years, after two consecutive negative tests with a one-year interval between them, for women aged 25 to 64 years.^4^ However, the age range and test interval recommendations are not widely followed.^6^ After several decades, the program has not achieved organized status, thus resulting in both low coverage and a lack of significant impact on mortality.^2^^,^^5^

The cities of Campinas (state of São Paulo, SP) and Curitiba (state of Paraná, PR), located respectively in the southeastern and southern regions of Brazil, are the administrative centers for two major regional areas with high human development indices, according to the World Health Organization (WHO).^7^ These regions have well-established public primary care networks, but there are no controls in place to monitor population screening for cervical cancer. The system is based on primary healthcare units that are connected to a tertiary-level regional hospital cancer center, to which cases of suspected high-grade intraepithelial lesions or cervical cancer are referred.

The Brazilian population-based cancer registry system started in São Paulo in 1969 and was then expanded to other major cities that support the Brazilian National Cancer Institute (Instituto Nacional do Câncer, INCA), which has been calculating cancer incidence since 1995.^5^ However, this registry system operates discontinuously and ineffectively in some places, including in the cities of Campinas, SP, and Curitiba, PR.^8^^,^^9^^,^^10^

These cities have regional hospitals that serve as the main reference for managing gynecological cancer among the residents of 82 municipalities in the Campinas region, covering 5.5 million people,^11^ and 95 municipalities in the Curitiba region, covering 5.1 million people.^12^ These two regions together represent around 5% of the Brazilian population. According to information from SUS regarding cervical cancer treatment, 97% of the cases in Campinas during the period 2010-2011 were managed at the Women’s Health Hospital (WH), which is located at the University of Campinas (Universidade de Campinas, UNICAMP). In Curitiba, for the period 2008-2012, 96% of the cases were managed at the Clinics Hospital (CH) of the Federal University of Paraná (Universidade Federal do Paraná, UFPR) and at the Erasto Gaertner Cancer Center Hospital (EGH).^8^^,^^9^^,^^10^

Although these regional hospitals do not record all cervical cancer cases, thus making it impossible to calculate the real incidence, their data enable estimation of cervical cancer incidence and trends in the populations covered by opportunistic screening, in regions of Brazil with comparatively high economic development.

## OBJECTIVE

The aim of the present study was to estimate the cervical cancer incidence rate (IR) and trends in the abovementioned Brazilian cities and their surrounding regions, for the period 2001-2012.

## METHODS

### Study design and setting

A population-based cohort study was conducted under the assumption that all cases of cervical cancer were managed in SUS cancer referral center hospitals. It can be expected that the vast majority of women with this neoplasia are treated at these centers. The hospitals considered were the Women’s Hospital (WH), in the Campinas region, and the Clinics Hospital (CH) and Erasto Gaertner Hospital (EGH), in the Curitiba region. The information about women with cervical cancer was collected from each hospital-based cancer registry system for the period from January 2001 to December 2012.

Only cases originating in municipalities that were located in each region, as defined by the official geographical distribution (political, administrative and/or healthcare system),^11^^,^^12^ and which systematically referred cases for care in the specified regional hospitals during the period 2001-2012, were considered.

### Patients’ records and general population

Records from 3,875 subjects were selected in accordance with the International Classification of Diseases (ICD-O) 2013 code C53.^13^ The histological types considered were squamous-cell carcinoma (SCC) and equivalents, adenocarcinoma (AC) or any variant thereof and adenosquamous carcinoma (ASC);^13^ 58 records with uncommon histological types that are less detectable through screening were excluded. Another 453 records relating to cases that arose from municipalities outside the official regions covered by these hospitals in Campinas and Curitiba were also excluded.^11^^,^^12^ Thus, a total of 3,364 records (1,646 from the Campinas region and 1,718 from the Curitiba region) were included for analysis.

The population base upon which the incidence estimates were drawn was the Brazilian official censuses conducted in the years 2000 and 2010, and yearly projections for the interval between them. The data were available according to gender and five-year age groups for each city considered. The detailed database for each city is accessible online for public and technical consultations.^14^^,^^15^

### Statistical analysis

Regional crude IRs were estimated using the number of cancer cases registered as the numerator and the female population for the same region and period as the denominator. To enable comparison between the regions studied, age-standardized incidence rates (ASRs) were obtained through a direct method, as described by the International Agency for Research on Cancer (IARC). These rates used the world standard population proposed by Segi in 1960.^16^ Trend analyses were done according to year and age group, using the same 25-64 age range that is used in the official Brazilian screening program.^4^ These analyses were also done according to histological type (using the WHO classification).^13^ The results were reported per 100,000 women per year and were presented in terms of the annual percentage change (APC) and as percentage points (pp). Trend analysis was done by means of linear regression using the StatsDirect statistical software (v. 3.0; StatsDirect, Cheshire, UK). Increasing or decreasing trends with P-values of less than 0.05 were considered statistically significant.

### Ethics

This study followed the recommendations of the National Health Council of Brazil and was previously approved by the ethics committee of each hospital, under the following approval numbers: UNICAMP 890.837, dated November 24, 2014; UFPR 1.031.168, dated March 30, 2015; and EGH 1.486.542, dated April 11, 2018.

## RESULTS

**Figure 1.**
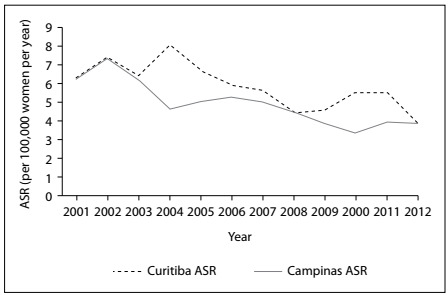
Estimated annual age-standardized incidence rate (ASR) for cervical cancer according to the region studied. The regions showed similar annual percentage change (APC) with decreasing trends (Campinas = ­0.29 pp, P < 0.001; Curitiba = ­0.26 pp, P = 0.003).

As shown in Table 1, the IR and ASR according to year were similar for the two regions. The ASRs for the two regions showed similar APCs with a decreasing trend, as can be seen in Figure 1 (Campinas = -0.29 pp, P < 0.001; Curitiba = -0.26 pp, P = 0.003).

**Table 1.** Cervical cancer incidence rate, estimated according to year and region

	Campinas region				Curitiba region			
Year	Population (N)	Cancer (n)	IR	ASR	Population (N)	Cancer (n)	IR	ASR
2001	2,385,508	141	5.9	6.2	2,227,430	132	5.9	6.3
2002	2,428,290	178	7.3	7.3	2,265,751	157	6.9	7.4
2003	2,472,054	148	6.0	6.2	2,307,103	141	6.1	6.4
2004	2,515,664	116	4.6	4.7	2,348,361	175	7.5	8.0
2005	2,614,776	127	4.9	5.0	2,442,000	152	6.2	6.7
2006	2,665,255	144	5.4	5.3	2,489,744	140	5.6	5.9
2007	2,727,899	155	5.7	5.0	2,538,530	146	5.8	5.6
2008	2,683,221	137	5.1	4.5	2,495,481	120	4.8	4.5
2009	2,717,171	125	4.6	3.9	2,526,281	128	5.1	4.6
2010	2,738,136	115	4.2	3.4	2,467,100	157	6.4	5.5
2011	2,767,814	127	4.6	4.0	2,488,139	159	6.4	5.5
2012	2,796,449	133	4.8	3.9	2,508,543	111	4.4	3.9

IR = crude incidence rate (per 100,000 women per year); ASR = age-standardized incidence rate (per 100,000 women per year).

**Figure 2.**
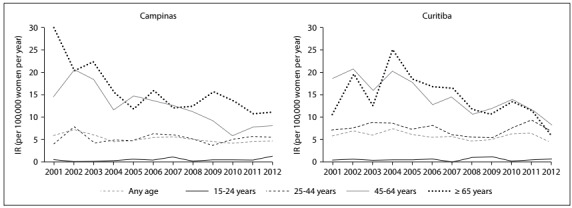
Annual evolution of the estimated incidence rate (IR) for cervical cancer per 100,000 women per year, according to age group and region.

Considering age-specific IR, the Campinas region exhibited progressive and substantial decreases in IR among women aged 45 years or older, with a decreasing APC trend of 1.05 pp for cancer incidence among women aged 45-64 years (P < 0.001) and 1.25 pp among women aged 65 years or older (P = 0.002) (Figure 2; Table 2). The same pattern was observed in the Curitiba region for the age range 45-64 years, for which a decreasing APC trend of 0.95 pp was observed (P < 0.001) (Figure 2; Table 2). For IR, the Campinas region showed an increasing APC trend of 0.06 pp in the age range 15-24 years (P = 0.046), with IR of 0.44 per 100,000 women in 2001 and 1.39 per 100,000 women in 2012. This effect was very small and is not visible in Figure 2. The other age groups did not show any significant trend in either region (Table 2).

**Table 2.** Crude cervical cancer incidence rate, estimated according to region for the years 2001, 2006 and 2012; and diagnosis trend over the period 2001 to 2012, according to age-specific group and histological type

	Incidence									
	Campinas region					Curitiba region				
	Rate according to year^a^			Trend^b^		Rate according to year^a^			Trend^b^	
	2001	2006	2012	APC	P	2001	2006	2012	APC	P
Age group^c^										
15-24 years	0.44	0.39	1.39	+0.06	0.046	0.47	0.63	0.70		0.655
25-44 years	4.10	6.15	5.59		0.962	7.17	8.07	6.71		0.507
45-64 years	14.75	13.69	8.02	-1.05	< 0.001	18.73	12.76	8.29	­0.95	< 0.001
≥ 65 years	29.96	15.98	11.13	-1.25	0.002	10.79	16.76	5.84		0.744
Histological type										
SCC	4.70	4.24	3.58	-0.17	0.001	5.39	4.86	3.79	­0.14	0.028
AC + ASC	1.22	1.16	1.18		0.988	0.54	0.76	0.64		0.457

^a^Incidence: number of cancer cases per 100,000 women per year.

^b^Trend for period 2001-2012 was presented as ”APC” (annual percentage change in percentage points): negative value indicates decreasing trend and positive indicates increasing trend.

^c^Age group: mean incidence in age-specific population. Number of cases under 20 years/15-24 years of age: 2/25 for Campinas region and 1/27 for Curitiba region.

Statistical test: simple linear regression.

SCC = squamous-cell carcinoma; AC = adenocarcinoma; ASC = adenosquamous carcinoma.

**Figure 3.**
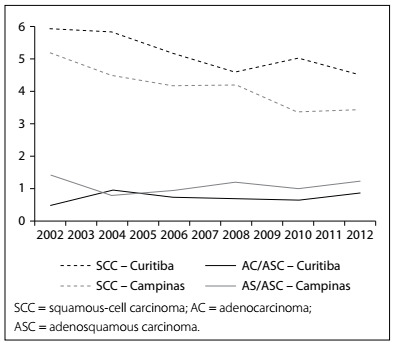
Estimated incidence rate for cervical cancer, according to histological type and region.

The histological type SCC presented a decreasing IR trend in both regions, with an APC of 0.17 pp in the Campinas region (P < 0.001) and 0.14 pp in the Curitiba region (P < 0.028), whereas the IRs for AC and ASC did not change over the period evaluated (Table 2; Figure 3).

## DISCUSSION

The annual cervical cancer IR and ASR estimates ranged from 3.9 to 8.0 per 100,000 women per year over the period 2001-2012, but were lower over more recent years. They were similar for the two regions. Longitudinal analyses revealed a significant and slowly decreasing trend that was similar to patterns identified in previous reports about cervical cancer incidence and mortality in Brazil.^2^^,^^3^

According to official information from the Brazilian population-based cancer registry that was released in 2018, INCA has calculated that the crude IR was 9.17 and the ASR was 6.97 in Campinas for the years 2010-2011. In Curitiba, the crude IR was 13.27 and the ASR was 10.82 for the years 2008-2012.^8^^,^^9^^,^^10^ According to an international report from ICO (2016), referring to the year 2012, the ASR per 100,000 women was 16.3 cases for Brazil, 14.2 cases for Campinas, 15.2 cases for São Paulo and 22.1 cases for Porto Alegre (in the south of Brazil).^17^ These rates were higher than the rates that we present here, and one possible explanation could be that cases from cities outside of the geographical areas of the present study, which were excluded here, were included in the incidence calculation in ICO (2016).

The slowly decreasing trends observed for IR and ASR in Campinas and Curitiba regions were similar to the IR patterns that have been reported from places that have made efforts to decrease cervical cancer rates, but without success in promoting changes to achieve organized screening programs. The classic example of a successful screening program comes from England, where, similarly to what was reported here, before 1988 the screening programs had low-impact on cervical cancer incidence. Cervical cancer rates started to change in England after implementation of strategies to organize the screening program, with IR decreasing faster, as seen from data reported in 1995, eight years after implementation of the modifications.^18^ In England, there was an 8% decrease in IR each year, from 16 cases to < 10 per 100,000. The main strategy used to achieve this decrease was the “call and recall” method, which was inserted in a system to register and control the screened population. Over the same period, the coverage of women screened increased from 42% in 1988 to 85% in 1994.^18^

Although the IR and ASR estimates of the present study may present some issues, the way in which the information originated from each region can be considered to have remained constant, which thus makes it possible to compare the longitudinal pattern of change in ASR. The ASR over the study period decreased by 35% (from 5.88 to 3.80) for the Campinas region and by 37% (from 6.96 to 4.37) for the Curitiba region. There was a clear reduction in the incidence of uterine CC over time, which was similar in the two regions studied. According to information available from INCA about ASR for the cities of São Paulo and Curitiba, comparing the period 2001-2005 with the period 2008-2012, there was a substantial decrease in ASR, as follows: São Paulo, 16.47 to 7.75 (-53%); Curitiba, 15.75 to 10.82 (-31%).^8^^,^^9^^,^^10^^,^^19^^,^^20^

The cancer cases registered were concentrated in the age group of 45 years or older, with higher estimated IR and an evident annual decreasing trend, which can be seen in Figure 2 for both regions (IR-2001/IR-2012: Campinas = 14.8/8.0; Curitiba = 18.7/8.3 for the age group 45-64 years; P < 0.001). It could be seen that the estimated IR decreased by around 50% over the years 2001-2012. A similar pattern was seen in the Netherlands over the period 1989-1998,^21^ thus showing the positive impact of organized screening, in that the number of cervical cancer cases decreased from 7.1 to 6.1 per 100,000 women per year, among which most cases were diagnosed at age 45 years or older.

As expected, the estimated IR in the 15-24 age group was very low, but the Campinas region exhibited an increasing trend, with IRs of 0.44 in 2001 and 1.39 in 2012 (per 100,000 women per year; two cases under 20 years of age and 23 cases in the 20-24 age range). There is controversy about the age at which screening should start, with some reports suggesting 18-21 years, as in United States and Australia,^22^^,^^23^ and others, including Canada, England and Brazil, advising that no screening should be done before the age of 25 years.^4^^,^^24^^,^^25^ In general, about 1% of all cervical cancer cases are diagnosed in women under 25 years old, and cervical cancer is considered rare before 20 years of age.^24^^,^^26^

In addition to the positive effects regarding estimated IR and ASR that were observed for specific age groups in the present study, the estimated IR for the SCC histological type of cervical cancer decreased. This can be considered to be another positive effect, although no change was observed for glandular histology. A similar pattern was reported in the Netherlands, as one of the first changes arising from implementation of organized screening over the period 1989-1998.^21^ A decreasing number of cervical cancer cases and an increasing proportion of glandular histological type can thus be considered as indications of effective screening through cytological tests. A recent report from Brazil with more than 50,000 cervical cancer cases registered over the period 2000-2009 showed an increasing proportion of glandular histological type (10% to 16%), but these are still considered modest values.^28^ The proportion of glandular histological type over the period 2001-2012 was 21%-26% for the Campinas region and 8%-16% for the Curitiba region.^29^

Our study presents some limitations. For example, only cases from referral hospitals within the public healthcare system were considered for calculating the estimated IR. However, there were no significant changes to the service network or any creation of new reference services for treatment of gynecological cancer during the period studied, and these hospitals (which were the data source) remained the main services treating women with gynecological cancer. Although the IR estimates may have had some bias, the trend analysis allowed assessments of changes in cervical cancer rates relating to opportunistic screening program.

We analyzed two regions of Brazil with prominent development, high human development indices, and established primary care networks, where at least 50% of the population is treated through the public health system (SUS), but where the only screening program is of uncontrolled and opportunistic nature. Therefore, our results probably included a high proportion of women who were outside the screening program and who received their cancer diagnoses after experiencing symptoms. Another report on the same population showed that 60% of the cases were diagnosed at advanced stages of the disease.^29^ This is an effect from opportunistic screening and is associated with low coverage of the target population. The number of cases of cervical cancer that were treated outside of SUS (and thus not analyzed here) can be considered minimal.

Some remarkable positive changes were observed in this study, including decreasing estimated IR among women over 45 years of age, and a decreasing trend for SCC histological type, perhaps reflecting the strong commitment by the regional healthcare system to control this cancer, albeit without organization. Although the results are slightly encouraging, these findings may give us an estimate of the best performance to be expected from opportunistic screening programs, with no tools to identify the screened and non-screened populations, similar to results reported by Costa et al.^5^

Hard work without a controlled screening program is unlikely to achieve the desired results, as shown here. Annually, SUS pays for enough Pap smears to cover 90% of the country’s total target population, despite the fact that 40 to 50% of this population is treated in private care networks. In the best-case scenario, therefore, the real coverage of cytological tests is only 30%, as previously reported for the city of Campinas.^6^

Additional financial support to combat this disease has produced limited results, as a consequence of the deficient organization of screening programs.^5^^,^^27^ Reaching an adequate level of organization is crucial for the future, including screening through new technologies or in vaccinated populations against HPV, and Brazil needs to promote effective changes in this process.

## CONCLUSION

Estimated IR and ASR for cervical cancer in two developed regions of Brazil showed a slowly decreasing trend, which was most evident among women aged 45 years or older and among cases with SCC histological type. These finding reflects the opportunistic nature of the population screening program, despite the comparatively high level of economic development in the two regions.
